# Damage Identification and Quantification in Beams Using Wigner-Ville Distribution

**DOI:** 10.3390/s20226638

**Published:** 2020-11-19

**Authors:** Andrzej Katunin

**Affiliations:** Department of Fundamentals of Machinery Design, Faculty of Mechanical Engineering, Silesian University of Technology, 44-100 Gliwice, Poland; andrzej.katunin@polsl.pl; Tel.: +48-32-237-1069

**Keywords:** damage identification, damage quantification, Wigner-Ville distribution, modal rotations, Euler-Bernoulli beam

## Abstract

The paper presents the novel method of damage identification and quantification in beams using the Wigner-Ville distribution (WVD). The presented non-parametric method is characterized by high sensitivity to a local stiffness decrease due to the presence of damage, comparable with the sensitivity of the wavelet-based approaches, however the lack of selection of the parameters of the algorithm, like wavelet type and its order, and the possibility of reduction of the boundary effect make this method advantageous with respect to the mentioned wavelet-based approaches. Moreover, the direct relation between the energy density resulting from the application of WVD to modal rotations make it possible to quantify damage in terms of its width and depth. The results obtained for the numerical modal rotations of a beam presented in this paper, simulating the results of non-destructive testing achievable with the shearography non-destructive testing method, confirm high accuracy in localization of a damage as well as quantification of its dimensions. It was shown that the WVD-based method is suitable for detection of damage represented by the stiffness decrease of 1% and can be identified and quantified with a high precision. The presented results of quantification allowed extracting information on damage width and depth.

## 1. Introduction

Modern maintenance and monitoring demands require detection, identification, and quantification of structural damage in the possible earliest stage of its initiation and propagation. For this purpose, numerous methods that use primarily non-destructive testing (NDT) techniques were developed in the last decades, which found application in damage identification and structural health monitoring (SHM) in mechanical, civil, military, and aerospace engineering, where enabling of a possibility of early damage detection and identification is of a crucial importance [1,2,3,4,5,6,7,8]. Many of the NDT and SHM approaches used for the structural assessment, especially for the large structures, are based on the analysis of structural modal parameters due to the possibility of easy and inexpensive measurements and effectiveness in damage identification.

Numerous early approaches in modal-based damage identification were based on the analysis of changes in natural frequencies [9,10,11,12] or whole frequency response functions [13,14,15], however, when the modal curvaturefors started to be taken into consideration, the sensitivity to damage presence was satisfactory enough to detect and identify small damage [16]. Nevertheless, the attempts to improve the sensitivity of the damage identification methods in the last decades resulted in the development of numerous algorithms that basically relied on the post-processing of raw vibration data. Many of these approaches were based on beams with a damage, which, in spite of their simplicity, still have numerous applications in mechanical and civil engineering.

The developed algorithms used to post-process raw modal curvatures used numerous mathematical formulations. In [17], Ratcliffe used finite difference approximation of the Laplacian operator to detect and localize damage in beams, which allowed identification of very small damage represented by a thickness reduction of 0.5%. The authors of [1] introduced curvature damage factor (CDF) in order to consider damage in multiple modal curvatures and presented the results of identification of a damage both for theoretical formulation of a beam and a real bridge. Messina et al. [18] introduced the Multiple Damage Location Assurance Criterion (MDLAC) to detect and localize damage in beam and frame structures based on the changes in natural frequencies, which was further improved by the authors of [19] by using a genetic algorithm. Lu et al. [20] identified damage in beams by determining differences in modal curvatures of intact and damaged beams using CDF proposed in [1]. The authors of [21] proposed a two-step damage identification algorithm which uses a damage index of strain statistical moment to localize damage in beam-type structures. Another damage index for damage localization was proposed in [22], which was based on the normalized uniform load surface curvature of tested structures. Silva and Maia [23] proposed an estimation of damage location using the errors determined from constitutive models of beams and demonstrated successful localization of the simulated damage starting from 10% of thickness reduction. Brasiliano et al. [24] proposed a baseline approach of damage detection and localization based on the residual error method, which implies subtraction of displacements of mode shapes of a damaged structure from the displacements of an intact one. A similar approach was also used by the authors of [25,26,27,28]. The improved version of this approach was reported by the authors of [29,30], where the adaptive baseline concept was proposed. These approaches, however, did not guaranteed proper localization and identification of small damage, which is essential in demanding NDT and SHM applications.

Numerous further attempts were focused on the application of wavelet transforms (WTs) due to their high sensitivity to small disturbances in the modal curvatures. The continuous wavelet transform (CWT) was successfully used by Gentile and Messina [31] for detection and localization of open cracks in beams. This approach was confirmed by Douka et al. [32] in the similar study of identification of cracks in beams, which was additionally improved by proposing an intensity factor law for the quantification of the crack size. CWT was successfully used by Rucka [33,34] to localize cracks with up to 5% of thickness reduction of a tested beam. Han et al. [35] adapted the wavelet packet transform for identification of damage in beam-type structures based on the proposed energy rate index. The approach based on CWT was applied to identification of cracks in a bridge beam-type structure [36], which justified its sensitivity to damage in practical applications. A similar study was performed by Bayissa et al. [37], where the authors used CWT for damage identification based on numerical data and then applied the proposed approach for identification of cracks in bridge structures. Montanari et al. [38] investigated the influence of minimizing the number of the measurement points of the mode shapes on the damage detectability using CWT and proposed an optimal sampling procedure for effective damage identification. The authors of [39] proposed the damage identification algorithm in beams using the discrete wavelet transform (DWT) which allows for reducing computational complexity of the post-processing procedures. This concept was enriched by Ren and Sun [40] by taking into consideration the metrics related to the wavelet entropy in order to enhance the damage detectability. Khorram et al. [41] applied CWT for identification and quantification of structural damage, however, the estimation of the damage size using WT-based approaches always depends on an applied wavelet, thus, cannot be generalized. The problem of dependency of the resulting damage signatures on the applied wavelet was investigated by Janeliukstis et al. [42,43], where the authors tested numerous wavelets and introduced a catalog of wavelets according to the introduced metric of the damage estimate reliability indicating sensitivity to damage, which partially resolved the problem. In order to overcome the mentioned problem with selection of parameters of applied wavelets in WT-based post-processing algorithms, several other methods were developed. For instance, the authors of [44,45] applied the fractal dimension (FD) concept, but the sensitivity to small damage was still unsatisfactory, i.e., the best results reported in these studies were obtained for 10% of a thickness reduction. Moreover, the FD-based approach is more demanding in terms of required number of samples in the modal curvatures [46].

The recent studies of structural damage identification in beams considered more sophisticated algorithms focused on the improvement of the damage detection and identification abilities. Although the WT-based post-processing algorithms reveal higher effectiveness in damage identification, besides the necessity of selection of an appropriate wavelet, they suffer from significant influence to numerical or experimental noise in modal curvatures, which require additional processing of modal curvatures or improving the algorithms by adding additional steps based on artificial intelligence or soft computing methods [47]. Several algorithms that allowed for overcoming the problem of environmental noise were developed the authors of [48,49], where they proposed a hybrid approach based on Teager energy operator and CWT able to identify damage under very noisy conditions and validated this approach in experimental studies. In another study reported by the same team of the authors [50], yet another method was proposed, which was based on the complex-wavelet approach allowing identification of cracks under the noisy conditions, however, the smallest damage was of 20% thickness reduction and its performance for small damage is unknown. The other ways of the detectability improvement reported to-date include a combination of WT with the principal component analysis [51] or optimization algorithms [52,53], but, in general, they need additional tuning of parameters of the algorithms, which make them difficult to generalize and implement in practical problems.

One of the solutions being considered to resolve the deficiencies of WT-based algorithms with tuning is to find an appropriate algorithm for a post-processing procedure which can allow retaining similar sensitivity to structural damage as the mentioned ones but eliminate the data dependency during selection of parameters of such algorithms. Since WT is an integral transform, it is evident to look for similar non-parametric transforms. One of such transforms is the short-time Fourier transform (STFT), which is one of the simplest integral time-frequency transforms (note that in the context of the considered class of problems we will further consider such a type of transform as the space-frequency one since in the modal-based damage identification problems the input signals are not the time series, but the distance variable). In several studies [54,55], the application of STFT for damage identification was reported, however, the direct application of this transform to the detection and localization of damage is not effective due to the lack of possibility to obtain good resolution both in space and frequency domains, which was confirmed in some studies [37,40].

An alternative approach is to select an integral transform which allows retaining the lack of necessity of selection and tuning of parameters and simultaneously retaining good resolution in the space-frequency domain. One of such a type of transform is the Wigner-Ville distribution (WVD), being the fundamental integral non-parametric transform from the Cohen’s class, similarly as STFT [56]. In spite of STFT, WVD is the quadratic transform with an optimal resolution and instantaneous power density spectrum in both considered domains [57]. Due to these properties, WVD found wide applicability primarily in the problems of technical diagnostics of rotary machinery and their condition monitoring [56,57,58,59,60], and in detecting defects in various applications [61,62,63]. To the best of the author’s knowledge, there were only several applications of WVD to the structural damage identification problems reported to date. Gillich and Praisach in their study [64] used WVD only for extraction the information on natural frequencies from the vibration signals, while Dai and He [65] applied WVD for the determination of damage position in the NDT technique based on ultrasonic guided waves.

The aim of this paper is to investigate the capability of the newly presented non-parametric damage identification algorithm based on WVD as well as to evaluate its performance in terms of the accuracy of damage identification and quantification of the damage based on the modal rotations obtained from numerical simulations. The simulations were prepared in such a way to simulate high-resolution data achievable from the shearography NDT method. The analysis of modal rotations is superior with respect to widely used modal curvatures as the input data, since it reveals higher sensitivity to damage, which was shown in several studies (see e.g., [24,66]), including the previous studies by the author of this paper [67,68]. In this study, this superiority will be shown for the newly presented post-processing algorithm. Moreover, the theoretical investigation on superiority of the WVD-based approach over STFT and WT is provided in the paper and the validation is performed by presenting the results of the case studies with the simulated damage in a beam.

## 2. Theoretical Background

The simplest transform used for the space-frequency analysis is the mentioned STFT proposed by Gabor [69], represented by the following integral equation:

(1) $$S\left(\tau ,\omega \right)=\int_{-\infty }^{\infty }s\left(x\right)w\left(x-\tau \right)e^{-i\omega x}dx,$$

where s(x) is the transformed signal in the space domain, w(τ) is the window function (usually the Gaussian window is assumed), τ is the space shifting factor of the window w, ω is the frequency, and S(τ,ω) is the complex function representing the result of transform in the form of phase and magnitude in the space-frequency domain.

As it was already mentioned, STFT operates with a fixed window in the space-frequency domain and changing the widow size in one domain results in a corresponding change in another domain. This property is connected with the uncertainty principle

(2) $$\Delta x\Delta \omega \geq \frac{1}{2},$$

where Δx and Δω are the bounds limiting the window in space and frequency domains, respectively.

Due to this limitation, STFT cannot provide appropriate resolution of S(τ,ω) in the space-frequency domain, thus cannot be effectively used for damage identification problems. To overcome this limitation, numerous researchers used CWT proposed in its present form by Goupillaud, Grossman and Morlet [70], and given by the following integral equation:

(3) $$W\left(a,b\right)=w\left(a\right)\int_{-\infty }^{\infty }s\left(x\right)\psi \left(\frac{x-b}{a}\right)dx,$$

where a and b are the scaling and translation parameters, w(a) is the weighting function usually set to $1/\sqrt{a}$ for energy conservation purposes, and $\psi \left(\frac{x-b}{a}\right)$ is the wavelet kernel, which defines the basis function of the transform.

Due to the possibilities of scaling and translation of the basis function, CWT has the property of the multiresolution analysis, which allows representing of W(a,b) in the space-scale domain in such a way that all the components of s(x) are well-localized in this domain, which makes it possible to effectively perform damage identification (proven in the numerous studies cited in Section 1). The main deficiency of application of CWT in such a class of problems is the necessity of selection of the basis function ψ, which has a crucial influence on the resulting representation of W(a,b), and thus, on the effectiveness of damage identification. Besides the question of the variable damage identification effectiveness, the difficulties occur when CWT results are used for damage quantification, since the magnitudes of the resulting wavelet coefficients depend on a selected basis function.

To solve this problem, it is proposed to apply WVD introduced by Ville [71] for applications in the problems related analysis of electrical signals. WVD is given by the following integral equation:

(4) $$WV\left(\tau ,\omega \right)=\int_{-\infty }^{\infty }f\left(\tau +\frac{x}{2}\right)f^{*}\left(\tau -\frac{x}{2}\right)e^{-2i\pi \omega x}dx,$$

where $f\left(\tau +\frac{x}{2}\right)f^{*}\left(\tau -\frac{x}{2}\right)$ is the instantaneous auto-correlation function, and WV(τ,ω) is the Wigner-Ville energy density spectrum of a transformed signal.

As one can observe, WVD is non-parametric, but retains most of properties useful from the point of view of damage identification applications. Moreover, as it can be observed from Equations (1) and (3), STFT and CWT has a similar origin and the difference between these transforms lies in the basis function, while the transformed signal is given in both cases in the explicit form. In contrast to this, in WVD the signal is not given in an explicit form, but is represented by the auto-correlation function, and enters twice into the integration operation, therefore WVD is a bi-linear transform [72] which belongs to the Cohen’s class of integral transforms. The WVD holds the properties of scaling and translation, similarly to CWT, which makes it useful for the numerous applications, including the damage identification problems investigated in this paper. These operations, although different than in CWT, where they are controlled by the parameters *a* and *b*, can be related to each other in both transforms (see [73] for more details). In spite of STFT and CWT, WVD is a non-linear (quadratic) transform which has the consequences in the resulting energy density spectra, namely, the appearance of cross-terms, which, in the case of damage identification problems, is the undesired property, since the cross-terms may mask the identified damage signatures. The WVD is directly connected with another distribution from the Cohen’s class—the Choi-Williams distribution (CWD) —given by the following equation:

(5) $$CW\left(\tau ,\omega \right)=\int_{-\infty }^{\infty }\int_{-\infty }^{\infty }\frac{e^{-\frac{u^{2}}{4x^{2}/\sigma }}}{\sqrt{4\pi x^{2}/\sigma }}f\left(\tau -u+\frac{x}{2}\right)f^{*}\left(\tau -u-\frac{x}{2}\right)e^{-2i\pi \omega x}du\;dx,$$

where CW(τ,ω) is the Choi-Williams energy density spectrum of a transformed signal, σ is the temporal scaling parameter, and u is the ambiguity domain parameter. From (5), one can extract the kernel function of the CWD in the following form:

(6) $$\Phi_{CW}\left(x,u\right)=e^{-\frac{{\left(2\pi xu\right)}^{2}}{\sigma }}.$$

For the case when σ→∞, $\Phi_{CW}\left(x,u\right)\to 1$, and (5) reduces to (4) [72].

Numerous comparative studies on the time-frequency representations obtained using the mentioned transforms can be found in [72,73]. The problem with cross-terms is also presented in the earlier works of one of the authors [74,75]. Nevertheless, the properties of WVD, including primarily the lack of parameters to tune and direct connection with the energy of the transformed signal, make it a good candidate for damage identification and quantification, which is the subject of the author’s studies presented in the next sections.

## 3. Analyzed Structure and Damage Scenarios

**Figure 1:**
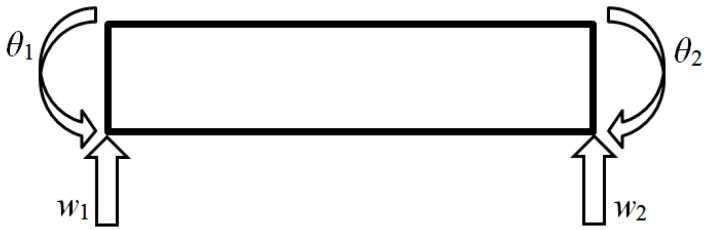
The two-node Euler-Bernoulli finite element marked displacements, $w_{1}$ and $w_{2}$, and rotations, $\theta_{1}$ and $\theta_{2}$.

**Figure 2:**
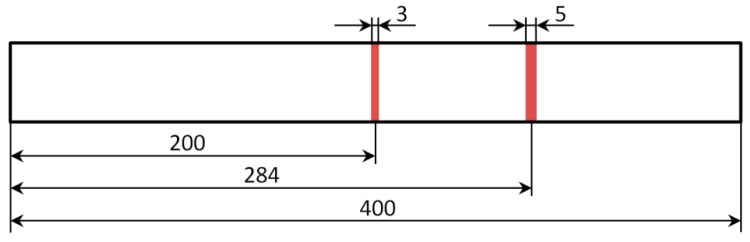
The geometry of the simulated beam with indication of the damage location.

**Figure 3:**
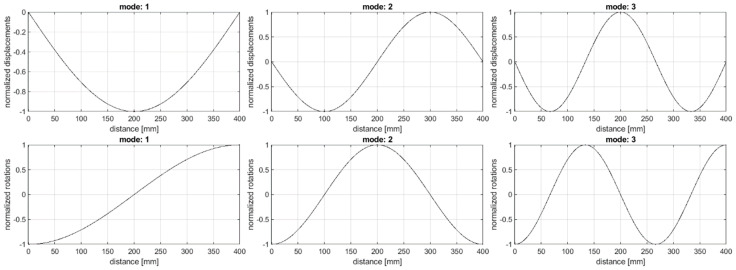
Displacements and rotations for the investigated modes.

In this study, the vibration of the Euler-Bernoulli beam was simulated with the finite element analysis to obtain the first three bending natural modes of vibration according to the studies reported in [76]. The aluminum beam model with the length of 400 mm, the width of 40 mm, and the thickness of 3 mm, and the following material properties: Young’s modulus of 67.8 GPa and the density of 2700 kg/m^3^, was discretized in 2263 equally spaced finite elements. The definition of such a dense finite element mesh is related to a possibility of obtaining high-resolution data using shearography, the optical NDT technique used by the author in damage identification studies in the previous works (see e.g., [77,78]). Each element was of 2 nodes, having two displacement and two rotation values (see Figure 1). The modal displacements and rotations were determined for the intact beam as well as for the damaged beam, considering the first damage in the form of a slot of the width of 5 mm and located at the distance of 284 mm and the second damage in the form of a slot of the width of 3 mm and located at the distance of 200 mm from the beam end (see Figure 2). For the damage identification study, eight damage scenarios were defined by combining the thickness reduction of the two slots, the respective values of which are shown in Table 1. Both displacements and rotations were collected as the results of simulation (see Figure 3).

In order to perform the damage quantification study, the same numerical model was used. A single damage in the form of slot with the width of 5 mm, located at the distance of 284 mm from the end of the beam, was simulated within seven scenarios with variable depth starting from 1% and ending with 20% of the thickness reduction, which are presented in Table 2.

## 4. Results of Damage Identification

In this study, the investigation of an application of WVD for damage identification and quantification was performed. The selection of WVD among the other available TFDs is due to the fact that WVD is the most energy-concentrated distribution within the distributions of the Cohen’s class [79]. According to the results of the initial studies, the application of CWD presented in Section 2 does not result in satisfactory results for small values of the temporal scaling parameter σ, typically used for calculations, due to much less sensitivity to the simulated damage as well as due to the appearance of cross-terms.

Both baseline-free and baseline approaches were presented in order to determine the sensitivity of the proposed damage identification method to simulated damage. Then, the analysis of sensitivity to damage when the displacements and rotations of a tested beam are taken into consideration as an input to the proposed processing procedures was investigated to show the superiority of the chosen modal rotations.

**Figure 4:**
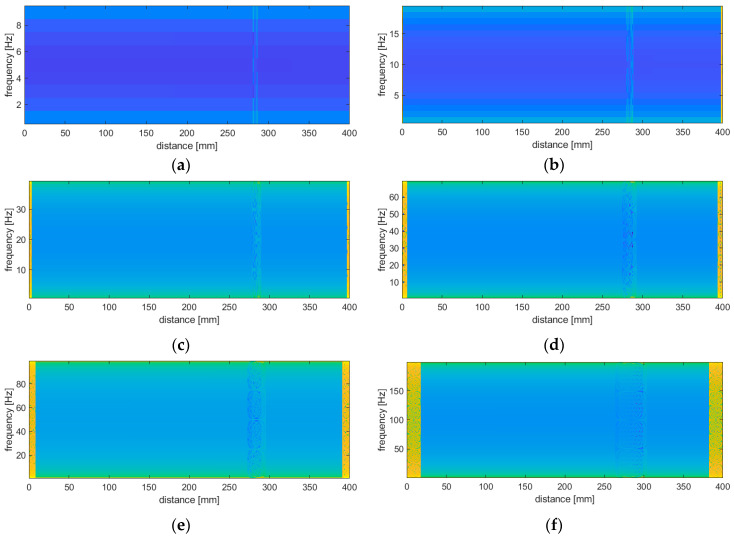
The influence of the frequency range on the damage detectability: the ranges of 0–10 Hz (**a**), 0–20 Hz (**b**), 0–40 Hz (**c**), 0–70 Hz (**d**), 0–100 Hz (**e**), and 0–200 Hz (**f**).

Although WVD is the non-parametric transform, as was already mentioned before, the influence of a frequency range onto the results of identified damage was observed. The increasing frequency range influencing on the increase of the width of the damage signature band in the resulting energy density distributions as well as on the appearance and widening of the side bands is known in the literature as the boundary effect. Several examples of the damage identification results for the damage scenario 3I and mode 1 are presented in Figure 4. To increase the visual distinguishability of the damage signatures the obtained energy values were raised to the power of 0.01. This value was selected based on empirical analysis, and it was found that the distinguishability of the damage signatures improved asymptotically with the decrease of a power. The assumed value seems to be appropriate, since its further decreasing does not affect any visual improvement of the damage signatures.

From the results presented in Figure 4, it is clearly visible that for the frequency ranges of 0–20 Hz and higher the damage localization and identification are not possible due to blurring the damage signature, while for the range of 0–10 Hz one can precisely localize the simulated damage and even identify its boundaries. Moreover, the boundary effect for this range of frequency does not appear, which is a great advantage of this approach with respect to WT-based damage identification procedures, where this effect can mask damage signatures near the boundaries as well as significantly decrease distinguishability of the detected damage, as it was reported in numerous studies [31,33,43,80]. One can observe that in the case of TFDs the frequency range is equivalent to the length of the support of the applied wavelet in the WT-based approaches in the light of the increasing the width of the distortion on the boundaries due to the boundary effect. Considering the above observations, the range of 0–10 Hz was assumed in the next studies.

### 4.1. Baseline-Free Approach

**Figure 5:**
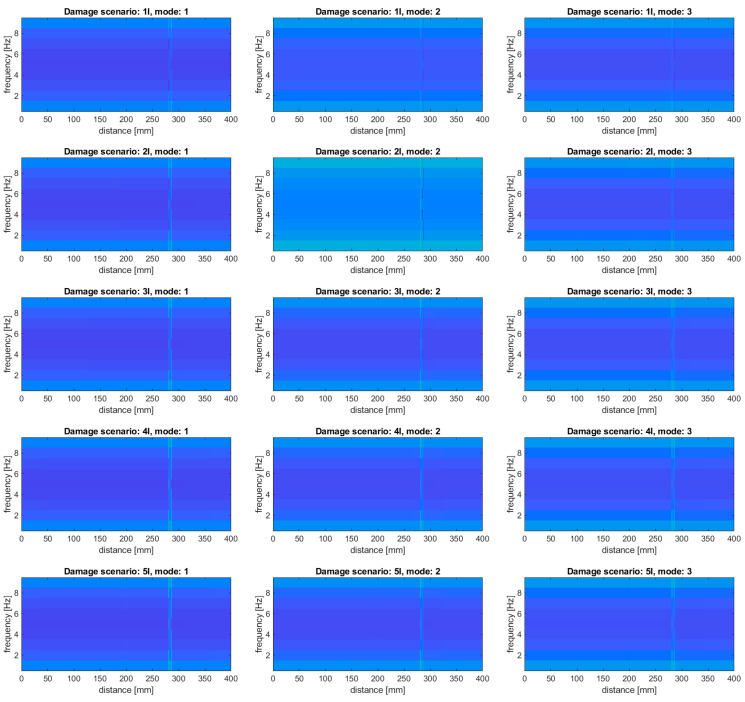

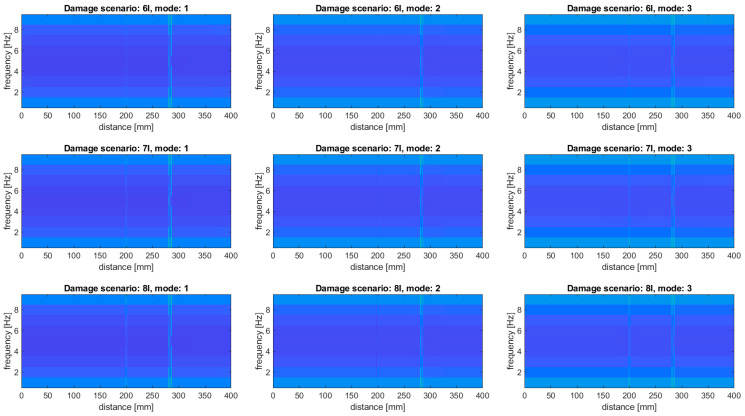
The results of the damage identification for the considered damage scenarios using a baseline-free approach.

The damage scenarios presented in Table 1 were used to investigate the detectability of the simulated damage in the form of slots for their various depths and locations. The obtained results for the particular damage scenarios and modes are presented in Figure 5.

From the obtained results, it can be noticed that only the second damage in the damage scenario 5I was not detected, while in the rest of the considered damage scenarios all the damage sites were well detectable and properly localized. It can be observed that the magnitude of energy density on the results presented in Figure 5 varies depending on the analyzed mode, which is especially well visible for the damage scenarios 6I–8I, where the damage signature for the second damage (located in the middle of the beam) is barely detectable for the second mode. This variability proves the proportional dependency of the resulting energy with the magnitude of vibration in the location of damage.

### 4.2. Baseline Approaches

In order to examine the possibilities of improvement of the ability of damage identification using the proposed WVD-based method, two baseline approaches were considered in this study. In general, the baseline approach was considered using of the modes of the intact beam as the reference data, however, the subtraction of the modal data for the damaged beam from the reference data can be performed in two ways: subtraction of mode shapes and then transforming the resulting differences and transforming the mode shapes for the intact and damaged beams separately and subtracting the resulting energy density spectra. According to the theoretical fundamentals of WVD [72], these operations are not interchangeable due to the appearance of the additional terms during subtraction of modes for the intact and damaged beams, thus the results of these baseline approaches will be different.

#### 4.2.1. Baseline Approach Based on the Transform of Differences

**Figure 6:**
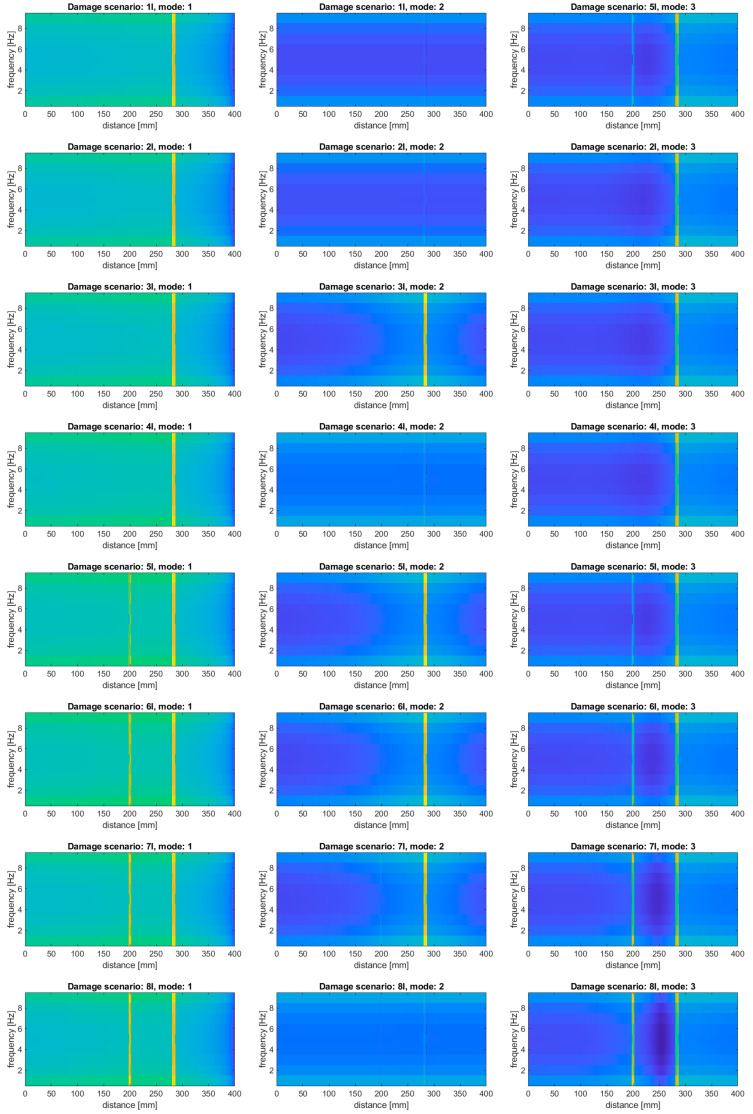
The results of the damage identification for the considered damage scenarios using baseline approach based on subtraction of the mode shapes before the transform.

The study was performed based on the results of subtraction of the mode shapes from the damage scenarios presented in Table 1 and the mode shapes on the intact beam. The results of the performed calculations are presented in Figure 6.

As it can be observed, the applied baseline-free approach makes it possible to identify all the simulated damage, including the damage scenario 5I, where the second damage became detectable. Moreover, the observed damage signatures are characterized by significantly higher distinguishability compared to the results of the baseline-free approach. However, in contrast to the results obtained using the baseline-free approach, the damage signatures observable in the obtained results using the presented baseline approach are blurred, which makes it impossible to identify the boundaries of the simulated slots.

#### 4.2.2. Baseline Approach Based on the Differences of the Transformed Modes

**Figure 7:**
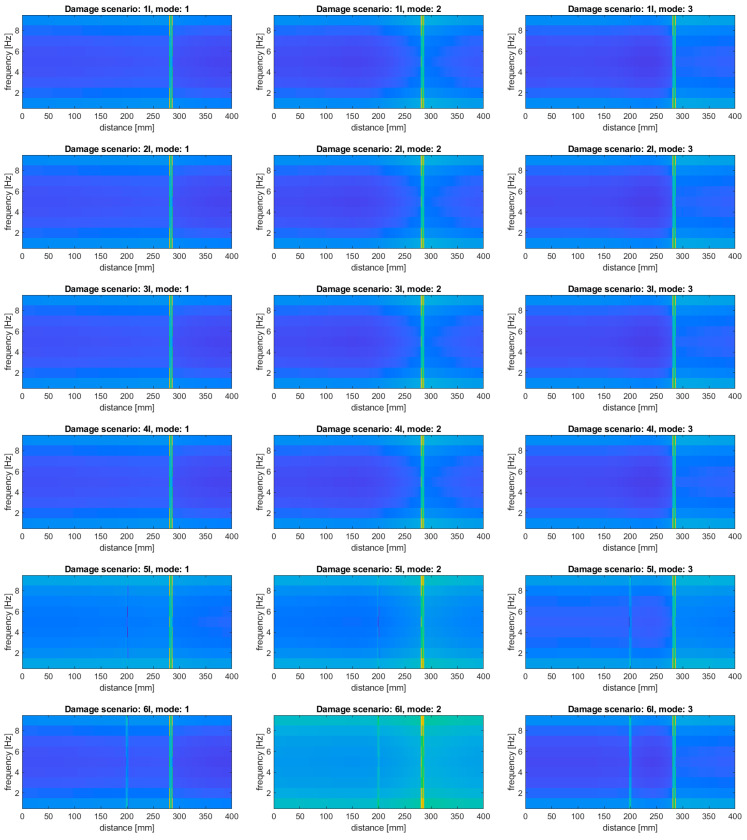

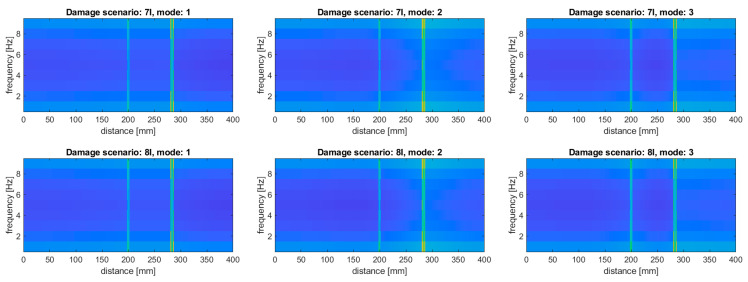
The results of the damage identification for the considered damage scenarios using baseline approach based on subtraction of the transformed mode shapes.

In this approach, the modes were subjected to transform using the proposed WVD-based method separately for the intact and damaged beams using the data for the defined damage scenarios presented in Table 1. The results of the calculations are presented in Figure 7.

Analyzing the results presented in Figure 7, one can notice that all the damage signatures in all the considered scenarios are well detectable. Comparing these results with the results obtained using the baseline approach based on transformation of mode differences, it can be stated that the performance of the following approach is better, since the distinguishability of the second damage, especially for the second mode in particular damage scenarios, is much better when the latter approach was used. Moreover, it can be observed that the boundaries of the simulated slots are well identifiable in the case of using the second baseline approach, which were undetectable in the most of damage scenarios when the first baseline approach was used. The superiority of the second baseline approach over the first one is justified theoretically, due to the lack of additional disturbances caused by transforming the differences of modes, which was discussed in the preamble to Section 4.2.

### 4.3. Displacements vs. Rotations

**Figure 8:**
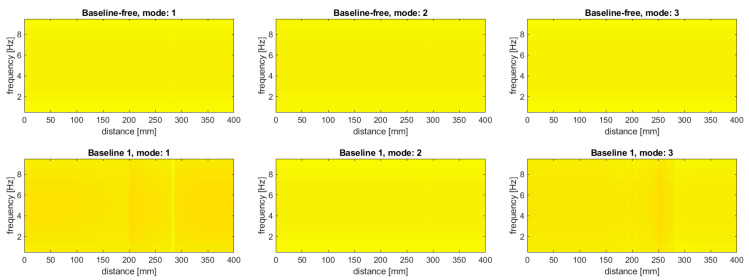

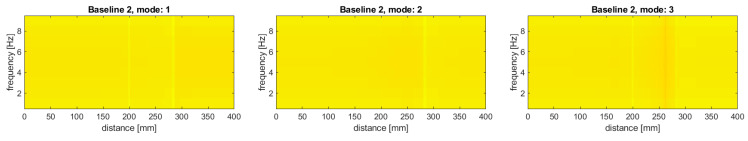
The results of the damage identification using modal displacements as the input data for the considered approaches.

In order to justify the selection of data for this study, namely, that modal rotations were selected instead of modal displacement, an additional study was performed. This justification was performed for the damage scenario 8I, where both simulated slots are well detectable with using modal rotations. The tests were performed for the considered baseline and baseline-free approaches, where the baseline approach based on the subtraction of the mode shapes before the transform was marked as “Baseline 1”, while the approach based on subtraction of the transformed mode shapes was marked as “Baseline 2”. The results for the modal displacements are presented in Figure 8.

It can be observed that, in general, the damage distinguishability is much lower compared to the results obtained for the modal rotations (cf. Figure 5, Figure 6 and Figure 7). The baseline-free approach, similar to the case of the modal rotations used as the input data, is characterized by the lowest sensitivity to damage. However, in contrast to the results obtained using modal rotations (see Figure 5), the simulated damage remains undetectable when the modal displacements were used. In the case of the baseline approaches, the simulated damage become to be barely detectable, excluding the results obtained for the second mode (both slots are undetectable for the “Baseline 1” approach, and the second slot is undetectable for the “Baseline 2” approach). Moreover, for both baseline approaches used for the third mode, additional disturbances appeared at the distance of 262 mm in the energy density spectra presented in Figure 8. These disturbances are probably the result of the application of WVD algorithm and can be considered as the cross-terms (see Section 2 for more details), which is undesirable due to the false damage signature appearance. Comparing the results obtained for modal displacements and rotations, one can conclude that the modal rotations provide better distinguishability of damage, since it is possible to obtain the derivatives of the mode shapes in a direct way, which is confirmed in the numerous independent studies (see e.g., [24,81]).

## 5. Results of Damage Quantification

**Figure 9:**
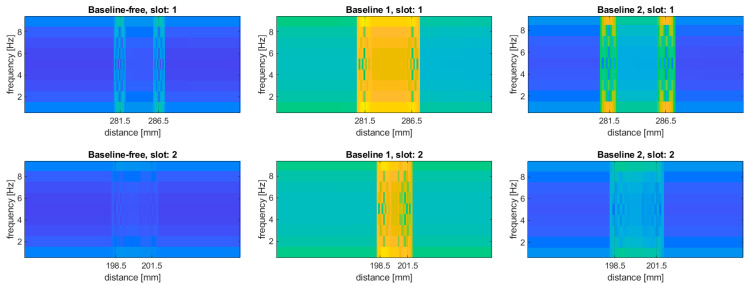
The exemplary results of the quantification of the damage width.

The quantification of damage was performed considering two dimensions of the simulated slots: their width and depth. From the previously presented results, it is possible to observe that for all the approaches considered in this study the difference in the width of the simulated slots is noticeable. In order to validate the width of the considered slots quantitatively, the analysis of the obtained results was performed. The exemplary magnifications of the damage signatures of the resulting energy density spectra obtained for the damage scenario 8I (mode 1) for all the considered approaches is presented in Figure 9. The widths of the identified slots were determined separately for the particular modes and damage scenarios and it was found that the determined width does not depend on the considered mode, neither on the damage scenario (i.e., depth of the slot). The results show that in all cases the identified damage signature is overestimated with respect to the true damage width, and this overestimation is of 1.53 mm and 0.71 mm for the slots 1 and 2, respectively. This implies the conclusion that the quantification error in this case depends on the damage width. However, the independency of the estimated width on the considered mode and the depth of the damage makes the proposed WVD-based method advantageous for further applications.

In order to quantify the damage depth, another dataset with elevated damage depths according to the scenarios presented in Table 2 was analyzed. The quantification was performed using all the approaches presented in this paper, namely, the baseline-free and two baseline ones. Since small variabilities in the values within the damage signatures are observable, both on the width of the damage signatures (due to the differences in energy density values for the detected boundary of a slot and the interior of the slot) and their length (due to the influence of the frequency), it is essential to consider that the energy density values corresponded with the boundaries of the detected damage. For this purpose, the values of the energy density from the locations of detected boundaries of the damage were averaged for a given frequency, and then the maximum value was taken from the collected values. The results of the depth quantification are presented in Table 3.

**Figure 10:**
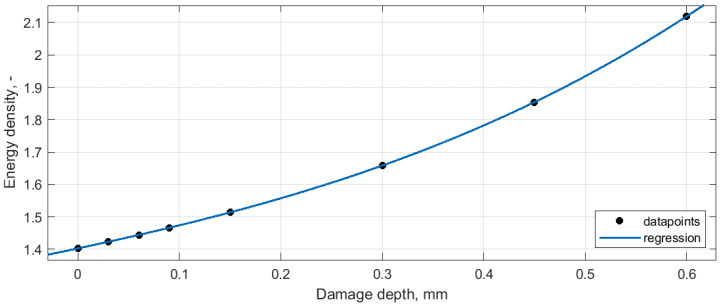
The exemplary result of the cubic polynomial regression of the quantification results for the mode 1 of the baseline-free approach.

The obtained values of the energy density for the investigated damage scenarios were subjected to the regression procedure in such a way that for particular approaches and particular modes the energy density values were plotted in a function on the increasing damage depth. This operation allowed to confirm the proportionality of the increase of the determined energy density values with respect to the considered damage depths. The cubic polynomial function was used for the regression due to the best fitting performance and the connection with the character of influence of a thickness reduction on the resulting structural stiffness. The estimation of the crack depth relationship with the local stiffness reduction based on polynomials is widely used in the literature (see e.g., [82,83,84,85]). The exemplary plot and the results of regression are presented in Figure 10. Note that the energy density for the intact beam was determined in the same way as for the damaged beams.

It was observed that the regression performance of the applied function allowed to obtain the exact relationship between the damage depth and the amount of the energy density. For all the considered approaches and all the considered modes, the coefficient of determination was of R^2^ = 1, which can be explained by the numerical character of the considered data. The obtained relationships allow for concluding that there exists a direct relation between the damage depth and the resulting energy density in the energy density spectra obtained using the WVD-based method. Following this, it is possible to quantify the damage depth based on comparison of the energy density values from the damage signature and damage-free regions. However, as it can be observed from the results presented in Table 3, the obtained energy density values for damage signatures are mode-dependent, which is a result of the various magnitudes of modal rotations for the particular modes. This means, that the operation of the quantification of the damage depth need to be performed for each considered mode separately.

## 6. Conclusions

The new method of damage identification and quantification based on the Wigner-Ville distribution has been presented to provide reliable information about location and size of single and multiple damage sites in beams. The presented algorithm is non-parametric, which means that the step of selection the parameters of processing typical for the WT-based approaches (such as selection of the wavelet function and its order) is omitted with retaining the sensitivity to damage comparable with the WT-based approach. Moreover, the proposed WVD-based method does not produce the boundary effect typical for WT-based approaches, and thus, allows identifying and quantifying damage also in the boundary regions. Three damage identification approaches were presented in this study: the baseline-free approach and two baseline approaches, which use the information of a condition of an intact structure. All the considered approaches reveal excellent sensitivity to damage, however the baseline approaches are characterized by considerably better distinguishability of the damage with respect to the baseline-free one, which is obvious due to the damage emphasizing by subtraction of the modes of damaged and intact structures. It was shown that the proposed method reveals high sensitivity to damage, i.e., for the baseline-free approach the detectability limit was of 0.1 mm of a thickness reduction, and for the baseline methods this limit was of 0.028 mm of a thickness reduction, which corresponds to 3.33% and 0.93%, respectively. It was also proven that using the modal rotations provides much better distinguishability of damage with respect to modal displacements, which was confirmed for all three considered approaches, both baseline and baseline-free. The performed damage identification studies proved that it is possible to identify the boundaries of a damage, while the results of the quantification studies show that the dimensions of a damage, namely its width and depth, can be accurately determined using the WVD-based method. The estimated damage width was insignificantly higher than the true width (1.53 mm for the 5 mm damage and 0.71 mm for the 3 mm damage), which seems to be acceptable from the point of view of practical applications, while the estimated damage depth was exactly the same as the true damage depth, which was proven by the exact matching regressions of the damage depth vs. energy density for all considered cases.

The above-presented advantages of the proposed WVD-based method creates a possibility of its application in practical problems and possible hardware implementation for the structural damage identification and SHM purposes. The performed studies were based on the data obtained from the numerical modal analysis of a beam in the form of modal rotations, and the influence of the measurement noise need to be investigated in order to evaluate the performance of this method and its validity for the application in practical problems. Nevertheless, the obtained results create a promising research direction for development of reliable non-parametric tools for NDT and SHM problems in mechanical and civil engineering. The proposed method can be applied to the experimental results obtained using the shearography NDT method, which among the high resolution and sensitivity to local structural changes can provide measurements of modal rotations in an explicit way.

## Figures and Tables

**Table 1:** The damage scenarios for the damage identification.

Scenario	1I	2I	3I	4I	5I	6I	7I	8I
Slot 1, mm	0.1	0.223	0.3	0.409	0.409	0.409	0.409	0.409
Slot 2, mm					0.028	0.1	0.2	0.3

**Table 2:** The damage scenarios for the damage quantification.

Scenario	1Q	2Q	3Q	4Q	5Q	6Q	7Q
Depth, mm	0.03	0.06	0.09	0.15	0.3	0.45	0.6

**Table 3:** The results of quantification of the damage depth.

Scenario		1Q	2Q	3Q	4Q	5Q	6Q	7Q
Baseline-free approach	Mode 1	1.42318	1.44410	1.46614	1.51391	1.65919	1.85357	2.11880
	Mode 2	0.31638	0.35909	0.40564	0.51167	0.87223	1.42781	2.28861
	Mode 3	0.01832	0.01945	0.02072	0.02373	0.03492	0.05389	0.08567
Baseline 1 approach	Mode 1	8.328·10^−4^	0.00346	0.00809	0.02433	0.11953	0.33466	0.75081
	Mode 2	9.535·10^−4^	0.00396	0.00927	0.02788	0.13711	0.38436	0.86364
	Mode 3	4.108·10^−5^	1.709·10^−4^	4.01·10^−4^	0.00121	0.00600	0.01702	0.03882
Baseline 2 approach	Mode 1	0.01986	0.04077	0.06281	0.11059	0.25586	0.45024	0.71548
	Mode 2	0.03917	0.08187	0.12842	0.23445	0.59502	1.15060	2.01139
	Mode 3	9.995·10^−4^	0.00213	0.00339	0.00641	0.01759	0.03656	0.06835
